# A study on the evaluation of competitiveness in the aviation logistics industry cluster in Zhengzhou

**DOI:** 10.1038/s41598-024-52697-x

**Published:** 2024-02-01

**Authors:** Zhihua Sun

**Affiliations:** Finance and Trade School of Zhengzhou, Shengda Economic and Trade Management University, Zhengzhou, 451191 Henan China

**Keywords:** Applied mathematics, Computational science, Computer science

## Abstract

As the global economy continues to evolve, air transportation is increasingly seen as a crucial factor in enhancing regional competitiveness. In particular, aviation logistics industry clusters have emerged as a new driving force for regional economic development. In this context, the current study aims to evaluate the competitiveness of the aviation logistics industry cluster in Zhengzhou, China. To achieve this goal, the study employs the “GEM model” and constructs a GKA evaluation model using evaluation index data from 21 logistics node cities across China in 2021. The entropy-weighted TOPSIS method is used for empirical analysis of the data. The results of the study reveal that the competitiveness of Zhengzhou’s aviation logistics industry cluster is moderately low. This is primarily due to the weak competitiveness of its foundational and regulatory subsystems. Specifically, the study finds that Zhengzhou’s resources, facilities, markets, government, and industry aspects are all less competitive when compared to other cities in China. In order to enhance the competitiveness of Zhengzhou’s aviation logistics industry cluster, the study recommends that efforts be made to improve the competitiveness of key elements such as resources, facilities, markets, and government. In particular, the focus should be on elevating industry competitiveness, followed by the development of appropriate regulatory strategies. By doing so, the aviation logistics industry cluster in Zhengzhou would be better positioned to compete with other clusters within China and globally.

## Introduction

As economic globalization progresses, air transportation has increasingly become a crucial means of enhancing regional competitiveness, making the aviation logistics industry a strategic point of breakthrough for regional economic development. Surveys have shown that aviation logistics have a strong ability to stimulate the economy and create jobs, with a revenue-generating ratio of up to 28 and an employment-driving ratio of 1:12^1^. Consequently, aviation logistics industry clusters have emerged as a new driving force for regional economic development.

Current research on logistics industry clusters, both nationally and internationally, primarily focuses on their definition, characteristics, influencing factors, formation mechanisms, development patterns, measurement methods, and competitiveness^2–6^.

Global supply chain management expert Sheffi identifies three types of optimized balances that lead to the formation of logistics industry clusters: logistics entity clusters, industry-university-research clusters associated with logistics, and service chain clusters that integrate logistics, commercial flows, information flows, cultural flows, and capital flows^7^.

Various evaluation models have been developed for assessing the competitiveness of logistics industry clusters, including the “Diamond” model, Growth, Engagement, and Monetization (GEM) model, and cloud model^6–8^.

Commonly used evaluation methods include principal component analysis, factor analysis, fuzzy comprehensive evaluation, DEA method, hierarchical analysis, and TOPSIS method^6–8^. National and international research on aviation logistics industries focuses on aviation logistics industries, aviation logistics parks, and aviation logistics industry competitiveness^9–13^.

Studies on the competitiveness of aviation logistics industries primarily focus on concepts, influencing factors, evaluation indicators, evaluation methods, and development paths^9–13^. After conducting a thorough literature review, several key findings and knowledge gaps have emerged that motivated the current research.

Firstly, the concept of a logistics industry cluster is still in its early stages of development, with no universally accepted definition due to its exploratory nature. Secondly, studies on the competitiveness of logistics industry clusters often utilize comparative evaluation methods, focusing on specific regions, cities, or ports. There is a relative scarcity of research evaluating the competitiveness of aviation logistics industry clusters. Lastly, most current research on Zhengzhou’s aviation logistics industry is qualitative, with limited empirical evaluation^14–17^.

This research, defines the logistics industry cluster as follows: A logistics industry cluster is a gathering of interconnected businesses, organizations, and infrastructure in a specific geographic area, all engaged in different aspects of logistics and supply chain management. This cluster usually comprises companies involved in transportation, warehousing, distribution, freight forwarding, customs brokerage, and related services. These clusters tend to form in particular regions due to advantageous geographic positioning, well-developed transportation infrastructure, a skilled workforce, and supportive government policies.

The cross-regional, cross-industry, and cross-departmental characteristics of logistics industry clusters result in comprehensive logistics systems that are formed by the interdependence and complexity of various logistics nodes and channels^18,19^. Therefore, the formation of aviation logistics industry clusters requires not only the construction of logistics parks and facilities but also support and promotion from hinterland cities. The contribution of this paper includes the following:

- Drawing on the experience of evaluating the competitiveness of port logistics industry clusters, this study focuses on 21 logistics node cities, including Beijing, Shanghai, and Zhengzhou.
- Based on the “GEM model,” the study constructs an aviation logistics industry cluster competitiveness Goal, Key results and Action (GKA) evaluation model, applies the entropy-weighted TOPSIS method for empirical evaluation of competitiveness, and presents conclusions and recommendations.

## Aviation logistics industry cluster competitiveness evaluation model

To comprehensively evaluate the competitiveness of aviation logistics industry clusters, a GKA evaluation model is employed. This model encompasses a systematic approach that involves three key steps:

- Firstly, an extensive indicator system is established, encompassing various aspects that contribute to the competitiveness of aviation logistics industry clusters. This system comprises indicators related to resource endowment, infrastructure development, market accessibility, government support, and industry performance.
- Secondly, the information entropy method is utilized to assign weights to each evaluation indicator. This method assesses the relative significance of each indicator, ensuring that the evaluation results accurately reflect the true competitiveness landscape.
- Finally, the entropy-weighted TOPSIS method is employed to evaluate the competitiveness of aviation logistics industry clusters. This method compares the performance of different clusters across the established indicator system and identifies the most competitive clusters, providing decision-makers with valuable insights.

By following these rigorous steps, decision-makers can gain a comprehensive understanding of the competitive landscape of aviation logistics industry clusters, enabling them to make informed policy and investment decisions that foster the growth and development of these crucial economic sectors^20–22^.

### GKA evaluation model

In 1988, Canadian scholars Tim Padmore and Hervey Gibson proposed the GEM model, which evaluates the competitiveness of industry clusters in a region. The model includes three key components: Grounding, Enterprises, and Markets. Grounding encompasses the resources and facilities available in the region, while Enterprises includes suppliers, related auxiliary industries, company structure, strategy, and competition. Markets involve both local and external markets. The GEM model has been widely used to evaluate the competitiveness of various industry clusters. As logistics industry policies are often formulated by governments, and the planning and construction of logistics infrastructure is a key factor in the development of logistics industry clusters, technological innovation is essential for improving logistics operation efficiency and updating logistics infrastructure^22–24^. Therefore, to better evaluate the competitiveness of aviation logistics industry clusters, the GEM model has been modified into the GKA model by incorporating subsystems coupling Grounding-Kernel-Adjust.

The GKA evaluation model is constructed around three interconnected subsystems: Grounding, Kernel, and Adjust.

The Grounding subsystem encompasses the fundamental pillars of an aviation logistics industry cluster, namely resources and facilities. This subsystem ensures the availability of essential assets and infrastructure that support the logistics operations. The Kernel subsystem focuses on the core competencies and capabilities of the cluster, represented by industry and innovation. This subsystem fosters the development of specialized logistics services, technological advancements, and a skilled workforce. The Adjust subsystem acts as the regulatory and mediating force, represented by markets and government. This subsystem ensures a favorable market environment, conducive government policies, and effective coordination between stakeholders. This modified GKA model explicitly recognizes the crucial role of government and technological innovation in shaping the competitiveness of aviation logistics industry clusters. It draws upon the pioneering research of Liu Youjin and Tang Fang, who established the foundation for this comprehensive evaluation framework.

**Figure 1:**
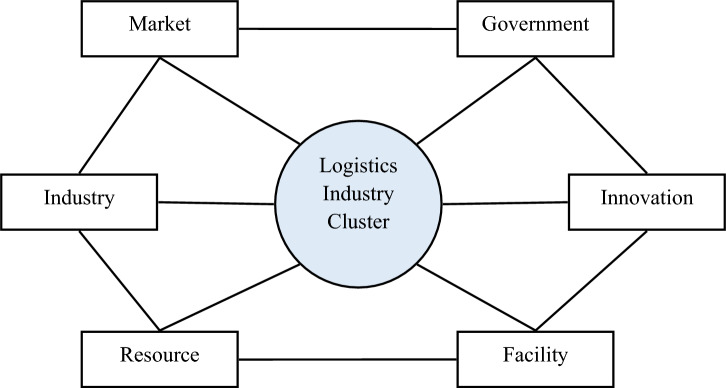
GKA evaluation model.

The GKA model offers a more nuanced and holistic approach to assessing the competitiveness of aviation logistics industry clusters. It provides valuable insights for policymakers and investors seeking to foster the growth and development of these dynamic economic sectors. Figure 1 depicts the GKA evaluation model, illustrating the interconnectedness of its three subsystems and their influence on overall competitiveness.

### Evaluation indicator system

The evaluation indicator system for the GKA model serves as a cornerstone of the framework for assessing the competitiveness of aviation logistics industry clusters. It is carefully constructed based on the characteristics of 21 prominent logistics node cities across the nation, adhering to the principles of data comparability, objectivity, availability, and completeness. The system encompasses a comprehensive set of indicators that are tailored to evaluate the competitiveness of aviation logistics industry clusters in each city. These indicators are organized into three interconnected subsystems: Grounding, Kernel, and Adjust.

The Grounding subsystem encompasses indicators that assess the fundamental pillars of the aviation logistics industry cluster, namely resources and facilities. This subsystem focuses on evaluating the availability and quality of essential assets, infrastructure, and support systems that underpin logistics operations.

The Kernel subsystem, on the other hand, concentrates on the core competencies and capabilities of the cluster, represented by industry and innovation. This subsystem evaluates the development of specialized logistics services, technological advancements, and a skilled workforce, which collectively determine the cluster’s ability to provide high-quality and efficient logistics solutions. The Adjust subsystem plays a crucial role in regulating and mediating the cluster’s environment, represented by markets and government. This subsystem evaluates the effectiveness of market mechanisms, the supportiveness of government policies, and the overall governance framework that fosters a conducive environment for logistics operations and industry growth.

**Figure 2:**
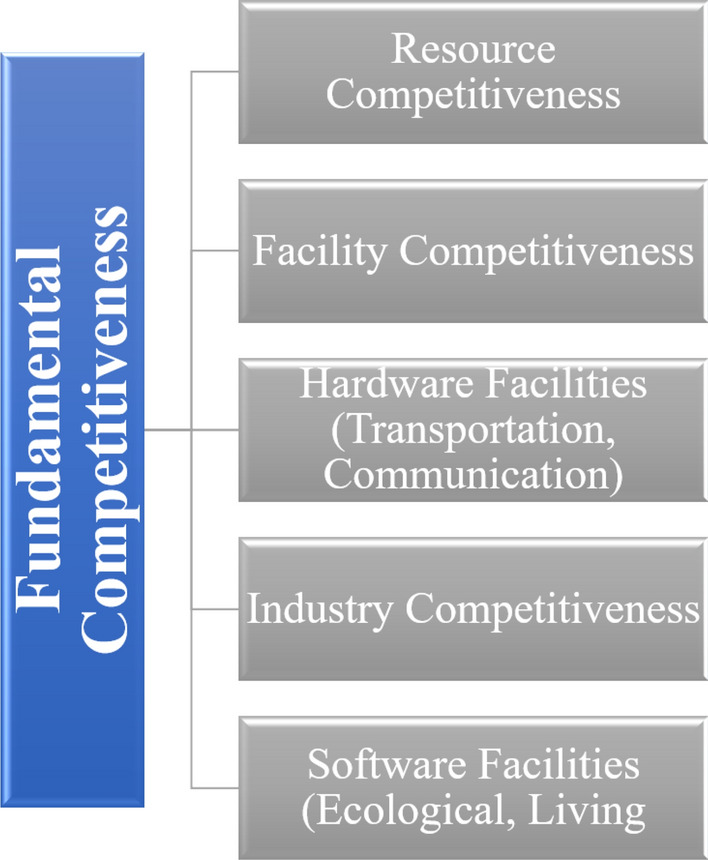
A conceptual framework for understanding the fundamental and core competitiveness of logistics industry clusters.

Figure 2 provides a conceptual framework for understanding the fundamental and core competitiveness of logistics industry clusters. This framework visually illustrates the distinct components that contribute to the overall competitiveness of these dynamic economic sectors.

The Adjust subsystem includes indicators related to markets and government. The Grounding subsystem includes four indicators: capital investment in logistics, transportation infrastructure, logistics facilities, and logistics personnel. These indicators measure the level of investment in logistics infrastructure and personnel in each city. The Kernel subsystem includes six indicators: logistics industry output value, logistics industry added value, logistics industry employment, logistics industry concentration, logistics industry innovation, and logistics industry competitiveness. These indicators reflect the overall performance of the logistics industry in each city. Finally, the Adjust subsystem includes five indicators: market size, market accessibility, market competition, government support, and government regulation. These indicators reflect the external environment and government support for the logistics industry in each city.

### Fundamental competitiveness

Fundamental competitiveness reflects the capacity of external factors in the logistics industry cluster to provide resources and facilities for internal logistics, manufacturing, and suppliers’ production and operation activities. Firstly, “resource competitiveness” is the foundation of its formation and development, including labor, capital, land, regional economy, and transportation location. Secondly, “facility competitiveness” is the prerequisite for its formation and development. Enterprises within the cluster rely on “facilities” to obtain “resources” and then carry out production and service activities. This includes hardware and software facilities. The hardware is manifested in transportation and communication facilities, reflecting the material conditions of its operation. The software is manifested in ecological and living facilities, reflecting the living, medical, and ecological environments for its development (see Table 1).

**Table 1:** Evaluation indicator system for the competitiveness of airport logistics industry clusters.

Objective layer (Z)	Subsystem layer (Z_*i*_)	Element layer (Z_*ij*_)	Variable layer (Z_*ijk*_)	Indicator layer (Z_*ijkw*_)
Competitiveness of airport logistics industry cluster (Z)	Grounding competitiveness (Z_1_)	Resource competitiveness (Z_11_)	Labor (Z_111_)	Number of employees (Z_1111_), Urban Registered unemployment rate (Z_1112_), Average number of on-post employees (Z_1113_), Average wage of on-post employees (Z_1114_)
			Capital (Z_112_)	Year-end financial institution deposit (z_1121_), Year-end financial institution loans (z_1122_), Actual utilization of foreign capital during the year (z_1123_)
			Land (Z_113_)	Total city area (Z_1131_), Urban area (Z_1132_), Built-up area (Z_1133_), Urban construction land area (Z_1134_), Proportion of urban construction land (Z_1135_)
			Regional economy (Z_114_)	Gross regional product (Z_1141_), Per capita gross regional product (Z_1142_), Gross regional product growth rate (Z_1143_), Fixed asset investment (Z_1144_)
			Transportation location (Z_115_)	Flight zone level (Z_1151_), Train station level (Z_1152_)
		Facility competitiveness (Z_12_)	Transportation facilities (Z_121_)	Aircraft takeoff and landing times (Z_1211_), Maximum aircraft model for takeoff and landing (Z_1212_), Parallel runway numbers (Z_1213_), Highway mileage (Z_1214_), Civilian car ownership (Z_1215_)
			Communication facilities (Z_122_)	Per capita postal and telecommunication services (Z_1221_), Internet users per 10,000 people (Z_1222_), Mobile phone users per 10,000 people (Z_1223_), Fixed telephone users per 10,000 people (Z_1224_)
			Ecological facilities (Z_123_)	Green coverage rate in built-up areas (Z_1231_), Industrial wastewater discharge (Z_1232_), Industrial smoke and dust emissions (Z_1233_), Comprehensive utilization rate of industrial solid waste (Z_1234_), Harmless treatment rate of national waste (Z_1235_)
			Living facilities (Z_124_)	Public buses per 10,000 people (Z_1241_), Taxis per 10,000 people (Z_1242_), Hospital beds per 10,000 people (Z_1243_), Per capita national water consumption (Z_1244_), Per capita national electricity consumption (Z_1245_), Per capita household gas consumption (Z_1246_), Public library collection per 100 people (Z_1247_)
	Core competitiveness (Z_2_)	Industry competitiveness (Z_21_)	Industry scale (Z_211_)	Postal service revenue (Z_2111_), Transportation, storage, and postal service employees (Z_2112_), Passenger traffic volume (Z_2113_), Freight traffic volume (Z_2114_), Express service volume (Z_2115_), Road passenger traffic volume (Z_2116_), Passenger throughput (Z_2117_), Road freight traffic volume (Z_2118_), Cargo and mail throughput (Z_2119_)
			Auxiliary industries (Z_212_)	Industrial electricity consumption (Z_2121_), Wholesale and retail employees (Z_2122_), Number of wholesale and retail enterprises above the designated size (Z_2123_), Total industrial output value of enterprises above the designated scale (Z_2124_), Number of industrial enterprises above the designated scale (Z_2125_), Total profits of industrial enterprises above the designated scale (Z_2126_)
			Industry advantages (Z_213_)	Passenger throughput growth rate (Z_2131_), Cargo and mail throughput growth rate (Z_2132_), Number of top 40 local general warehousing enterprises in the country (Z_2133_), Number of top 50 local logistics enterprises in the country (Z_2134_)
		Innovation competitiveness (Z_22_)	Technological innovation (Z_221_)	Number of college students per 10,000 people (Z_2211_), Full-time teachers in ordinary higher education institutions (Z_2212_), Per capita expenditure on science and education (Z_2213_), Proportion of science and education expenditure to gross regional product (Z_2214_), Science and education industry employees (Z_2215_), Patent applications granted (Z_2216_), Technology contract transaction amount (Z_2217_)
	Regulatory competitiveness (Z_3_)	Market competitiveness (Z_31_)	Local market (Z_311_)	Total retail sales of consumer goods (Z_3111_), Total sales of wholesale and retail goods above the designated size (Z_3112_)
			External market (Z_312_)	Total import and export volume (Z_3121_), Number of inbound tourists received (Z_3122_), International tourism foreign exchange income (Z_3123_), Proportion of output value of industrial enterprises above the designated scale with foreign investment (Z_3124_)
		Government competitiveness (Z_32_)	Fiscal support (Z_321_)	Public fiscal expenditure (Z_3211_), Urban maintenance and construction fund expenditure (Z_3212_), Fiscal expenditure on transportation (Z_3213_)

*Data Source* Author’s Compilation.

### Core competitiveness

Core competitiveness is determined by the scale structure, competitive advantages, and innovation capabilities of logistics enterprises and suppliers within the logistics industry cluster. Firstly, “industry competitiveness” includes industry scale, auxiliary industries, and industry advantages. The logistics industry can provide auxiliary services to other industries (or sectors) with high penetration and relevance. Therefore, the key to a logistics industry cluster gaining lasting competitive advantages lies in having first-class suppliers and close cooperation between upstream and downstream auxiliary industries. The scale of the logistics industry represents its capacity to provide logistics services to other industries, while the scale of auxiliary industries indicates its demand capacity for the logistics industry. The two complement and couple each other to form competitive advantages, ultimately promoting the formation of the logistics industry cluster. Thus, the three together constitute the foundation of core competitiveness^22–26^.

Secondly, the competitive advantage of the logistics industry cluster lies in the quality of logistics services, which depends on the support of modern technology. The main manifestations are as follows: Firstly, the rapid development of e-commerce promotes the widespread application of technologies such as the Internet of Things, cloud computing, and big data, shortening the distance between enterprises, facilitating cooperation and frequent information sharing. This generates a wide range of personalized logistics demands, driving the formation and development of logistics industry clusters. Secondly, advances in technology such as e-commerce, big data, artificial intelligence, and vehicle networking accelerate the construction of logistics information system platforms, promote the intelligent and networked development of logistics equipment, and improve the efficiency of logistics industry and supply chain operations. Therefore, technological innovation constitutes the “engine” of core competitiveness^23–27^.

## Regulatory competitiveness

Regulatory competitiveness refers to the ability of the market and government to influence the formation and development of logistics industry clusters.

Market competitiveness encompasses both local and external markets. Local market competitiveness drives the growth of logistics industry clusters by influencing the flow of consumer goods, capital goods, labor, capital, and technology. External market competitiveness plays a complementary role, facilitating the exchange of resources and products between markets.

Government competitiveness, on the other hand, provides the necessary infrastructure and policies that support the development of logistics industry clusters. Government investments in transportation, communication, ecological, and living facilities directly impact regional economic growth and transportation location advantages, laying the foundation for logistics cluster development.

**Figure 3:**
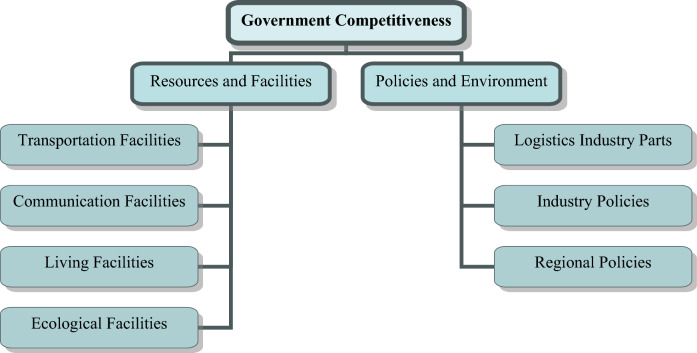
A conceptual framework for understanding the components of government competitiveness.

Furthermore, government planning of logistics industry parks guides the rational flow and agglomeration of production factors, directly influencing the formation of clusters. Government policies, such as industry, land, credit, employment, and regional policies, as well as measures like price control, tax subsidies, administrative approval, anti-monopoly, anti-unfair competition, and market supervision, shape the scale, structure, and competitive advantages of logistics industry clusters. Figure 3, shows a conceptual framework for understanding the components of government competitiveness, which is one of the factors that contribute to the regulatory competitiveness of logistics industry clusters.

Figure 3 shows a useful conceptual framework for understanding the different components of government competitiveness that contribute to the regulatory competitiveness of logistics industry clusters. By identifying the key resources, facilities, and policies that governments can use to support the development of logistics industry clusters, the framework can help policymakers and industry stakeholders to develop strategies for promoting the growth and competitiveness of the logistics industry.

### Entropy weight method

In 1948, Claude Elwood Shannon first introduced the concept of entropy in information theory. Information entropy refers to the uncertainty of the source signal in the communication process. The entropy weight method is an objective weighting method, where the greater the indicator’s information entropy, the smaller its variation coefficient, and the less information it provides, the smaller its role in comprehensive evaluation and the smaller its weight^20^. The calculation steps are as follows:

### Establish a standardized evaluation matrix

As shown in Table 1, assuming that the logistics industry cluster competitiveness system consists of subsystem, element, variable, and indicator layers, and that the indicator layer consists of *m* objects and *n* evaluation indicators, an evaluation matrix $X = (x_{ij} )_{m \times n}$ can be constructed and dimensionless processed to obtain a standardized decision matrix $X^{\prime} = (x^{\prime}_{ij} )_{m \times n}$, where the equation of $x^{\prime}_{ij}$ is^32^:

1 $$x^{\prime}_{ij} = \frac{{x_{ij} - \mathop {\min x_{ij} }\limits_{1 \le j \le n} }}{{\mathop {\max x_{ij} }\limits_{1 \le j \le n} - \mathop {\min x_{ij} }\limits_{1 \le j \le n} }}.$$

In the equation, *i* and *j* represent the row and column index of element *x*_*ij*_ in the matrix. Also, $\mathop {\max x_{ij} }\limits_{1 \le j \le n}$ and $\mathop {\min x_{ij} }\limits_{1 \le j \le n}$ are the maximum and minimum values of *j*, clearly, $0 \le x^{\prime}_{ij} \le 1$.

### Apply information entropy to assign weights

Calculate the information entropy *R*_*j*_ of each indicator according to $X^{\prime} = (x^{\prime}_{ij} )_{m \times n}$^33^:

2 $$R_{j} = \sum\limits_{i = 1}^{m} {u_{ij} } \ln u_{ij} ,\;i = 1, \ldots ,m; \, j = 1, \ldots ,n,$$

where *u*_*ij*_ represent the probability of presence of *x*_*ij*_ in matrix *x*, and is calculated as follows^33^:

3 $$u_{ij} = \frac{{1 + x^{\prime}_{ij} }}{{\sum\limits_{i = 1}^{m} {(1 + x^{\prime}_{ij} )} }}.$$

Calculate the variation coefficient *S*_*j*_ of each indicator according to information entropy *R*_*j*_:

4 $$S_{j} = 1 - R_{j} ,\;j = 1, \ldots ,n.$$

It is evident that the smaller the information entropy *R*_*j*_, the larger its variation coefficient *S*_*j*_, and the more information it provides, i.e., the greater the weight of the *j* indicator; otherwise, the smaller the weight of the indicator. Therefore, the information entropy weight *w*_*j*_ of the *j* indicator is:

5 $$w_{j} = \frac{{S_{j} }}{{\sum\nolimits_{j = 1}^{n} {S_{j} } }} = \frac{{1 - R_{j} }}{{n - \sum\nolimits_{j = 1}^{n} {R_{j} } }}.$$

### Entropy-weighted TOPSIS evaluation method

In 1981, C.L. Huang and K. Yoon first proposed the “Technique for Order Preference by Similarity to Ideal Solution” (TOPSIS), which has now become a widely used multi-objective decision-making method in systems engineering. The TOPSIS method, which involves determining an optimal positive ideal solution and a worst negative ideal solution, followed by calculating the Euclidean distances between each alternative solution and these ideal solutions, ranking them, and determining their merits, is a widely used method for evaluating alternatives. Wei and Guocai^22^ extended the TOPSIS method by combining it with the entropy weight method, creating the entropy-weighted TOPSIS evaluation method. This enhanced the objectivity of the evaluation process and the credibility of the evaluation results.

### Calculate Euclidean distances

*Step 1* Determine the positive and negative ideal solutions of the standardized decision matrix of the indicator layer. Select the highest and lowest values of each indicator in $X^{\prime} = (x^{\prime}_{ij} )_{m \times n}$ as the positive ideal solution *X*^+^ and the negative ideal solution *X*^−^:

6 $$X^{ + } = \left( {\mathop {\max x^{\prime}_{i1} ,}\limits_{1 \le i \le m} \mathop {\max x^{\prime}_{i2} ,}\limits_{1 \le i \le m} ...,\mathop {\max x^{\prime}_{in} }\limits_{1 \le i \le m} } \right),$$

7 $$X^{ - } = \left( {\mathop {\min x^{\prime}_{i1} ,}\limits_{1 \le i \le m} \mathop {\min x^{\prime}_{i2} ,}\limits_{1 \le i \le m} ...,\mathop {\min x^{\prime}_{in} }\limits_{1 \le i \le m} } \right).$$

According to (6), *X*^+^ is a vector representing the maximum value of each indicator, while *X*^−^ in (7) represents the minimum value of them.

*Step 2: Calculate Euclidean distances* By using the entropy weight method to assign weights to indicators and using the Euclidean distance formula for weighted average calculation, the distances and between each city in the indicator layer and the positive ($d_{i}^{ + }$) and negative ($d_{i}^{ - }$) ideal solutions can be obtained^34^.

8 $$d_{i}^{ + } = \sqrt {\sum\limits_{j = 1}^{n} {w_{j} (x^{\prime}_{ij} - x_{j}^{ + } )^{2} } } ;\;i = 1,2, \ldots ,m;\;0 \le d_{i}^{ + } \le 1$$

9 $$d_{i}^{ - } = \sqrt {\sum\limits_{j = 1}^{n} {w_{j} (x^{\prime}_{ij} - x_{j}^{ - } )^{2} } } ;\;i = 1,2, \ldots ,m;\;0 \le d_{i}^{ - } \le 1$$

In the equations, the indicator weight vector is determined by the entropy weight method $W = (w_{1} ,w_{2} ,...,w_{n} )^{T}$.

### Calculate proximity and rank

*Step 1* Calculate the proximity of each city in the indicator layer.

Based on the two distances $d_{i}^{ + }$ and $d_{i}^{ - }$, calculate the proximity $\eta_{i}$ of each city^35^:

10 $$\eta_{i} = \frac{{d_{i}^{ - } }}{{d_{i}^{ - } + d_{i}^{ + } }};\;i = 1, \ldots ,m;\;0 \le \eta_{i} \le 1.$$

The larger $\eta_{i}$, the more ideal the city’s status, indicating that city *i* is closer to the ideal value in the evaluation indicators, representing higher competitiveness. If all evaluation indicators of a city are positive ideal solutions, then $\eta_{i} = 1$; otherwise, $\eta_{i} = 0$.

*Step 2* Rank and compare the proximity of cities in the indicator layer.

Rank all city proximities $\eta_{i}$ to obtain single-level ranking, and then compare the competitiveness of each city’s logistics industry cluster in the indicator layer based on this ranking.

### Determine the competitiveness of all city aviation logistics industry clusters

Since the competitiveness of logistics industry clusters is a multi-layered construct, encompassing subsystem, element, variable, and indicator levels (as shown in Table 1), a multi-level comprehensive evaluation approach is necessary. This involves iterating through the entropy weight assignment and entropy-weighted TOPSIS evaluation steps multiple times.

At each level, the entropy weight method is used to determine the weights of the comprehensive indicators in the previous level, and the TOPSIS method is employed for comprehensive evaluation. The proximities of the previous level in each region are then used to create the evaluation matrix data for the next level, and the process is repeated for reassignment, calculation, and ranking.

This iterative process ensures that the evaluation captures the nuances of each level and provides a comprehensive understanding of the overall competitiveness of aviation logistics industry clusters^26–30^.

#### Empirical evaluation of zhengzhou aviation logistics industry cluster competitiveness

##### Evaluation index data processing

The data were collected through data sources such as “2022 China Urban Statistical Yearbook”, “2022 China Logistics Yearbook”, “2021 Civil Aviation Airport Production Statistical Bulletin”, etc. The data were calculated and organized in form of indicators listed in Table 1 for 21 national logistic node cities. Then, since some of the original indicators such as flight zone level, train station level, and maximum takeoff and landing aircraft type are qualitative; Then they were converted into quantitative indicators based on their rank order. To do this, first a list of unique values of each indicator was constructed, and then, the values of unique list were ordered by their order and the ordered values were assigned with natural ascending numbers. Finally, the values of quantitative indicators were replaced by the corresponding numbers. As some indicators are negatively correlated with the competitiveness of logistics industry clusters, such as the urban registered unemployment rate, industrial wastewater discharge, and industrial dust emissions, they were converted into inverse indicators^29–31^.

##### Entropy-weighted TOPSIS evaluation results and analysis

Based on the 2021 indicator data of 21 national logistics node cities, MATLAB 7.0 software is used to apply the entropy-weighted TOPSIS method for layer-by-layer evaluation of their logistics industry cluster competitiveness, with the results shown in Tables 2, 3, and 4.

**Table 2:** Competitiveness of aviation logistics industry clusters at the variable layer for national logistics node cities.

City	Labor		Capital		Land		Regional economy		Transportation location		Transportation facilities		Communication facilities		Ecological facilities	
	*η*_*i*_	Rank	*η*_*i*_	Rank	*η*_*i*_	Rank	*η*_*i*_	Rank	*η*_*i*_	Rank	*η*_*i*_	Rank	*η*_*i*_	Rank	*η*_*i*_	Rank
Beijing	0.899	1	0.718	2	0.503	2	0.594	3	1.000	1	0.696	1	0.316	4	0.604	2
Wuhan	0.171	14	0.219	9	0.274	9	0.481	9	0.690	2	0.283	13	0.174	10	0.506	9
Guangzhou	0.324	7	0.327	5	0.427	5	0.583	5	1.000	1	0.520	5	0.254	5	0.489	12
Shanghai	0.622	2	0.743	1	0.494	3	0.589	4	1.000	1	0.606	2	0.236	6	0.461	17
Nanjing	0.281	8	0.211	10	0.258	10	0.471	10	0.690	2	0.287	12	0.170	11	0.471	16
Zhengzhou	0.190	13	0.129	13	0.329	7	0.368	14	0.690	2	0.296	10	0.153	17	0.484	13
Shenzhen	0.620	3	0.413	4	0.438	4	0.604	2	0.501	3	0.432	7	0.641	1	0.478	15
Jinan	0.205	11	0.079	17	0.130	19	0.444	11	0.690	2	0.270	17	0.124	19	0.535	7
Lanzhou	0.148	17	0.004	20	0.128	20	0.278	20	0.499	4	0.028	21	0.067	21	0.456	18
Xi’an	0.149	15	0.146	12	0.173	14	0.338	15	1.000	1	0.455	6	0.157	15	0.631	1
Tianjin	0.280	9	0.524	3	0.346	6	0.633	1	0.690	2	0.394	9	0.166	13	0.446	20
Chengdu	0.360	5	0.243	8	0.316	8	0.419	13	1.000	1	0.526	3	0.202	7	0.524	8
Hangzhou	0.439	4	0.270	7	0.179	12	0.509	7	0.501	3	0.399	8	0.195	8	0.452	19
Chongqing	0.325	6	0.302	6	0.621	1	0.558	6	0.690	2	0.520	4	0.376	2	0.429	21
Xiamen	0.219	10	0.049	18	0.175	13	0.327	17	0.310	5	0.265	18	0.321	3	0.588	4
Urumqi	0.108	19	0.002	21	0.144	18	0.278	19	0.310	5	0.256	20	0.155	16	0.545	6
Qingdao	0.149	16	0.149	11	0.199	11	0.503	8	0.690	2	0.291	11	0.163	14	0.552	5
Dalian	0.106	20	0.082	16	0.153	16	0.334	16	0.310	5	0.275	15	0.108	20	0.490	11
Shenyang	0.078	21	0.085	15	0.156	15	0.063	21	0.690	2	0.277	14	0.125	18	0.505	10
Ningbo	0.197	12	0.109	14	0.095	21	0.428	12	0.310	5	0.271	16	0.167	12	0.481	14
Nanning	0.132	18	0.042	19	0.145	17	0.284	18	0.690	2	0.260	19	0.190	9	0.590	3
Mean	0.286		0.231		0.271		0.433		0.664		0.362		0.212		0.510	
Standard Deviation	0.208		0.214		0.150		0.144		0.240		0.152		0.123		0.057	
Range	0.821		0.741		0.526		0.570		0.690		0.668		0.574		0.202	
Coefficient of Variation	0.726		0.926		0.556		0.332		0.361		0.418		0.580		0.111	

City	Living facilities		Industry scale		Auxiliary industries		Industry advantages		Technological innovation		Local market		External market		Fiscal support	
	*η*_*i*_	Rank	*η*_*i*_	Rank	*η*_*i*_	Rank	*η*_*i*_	Rank	*η*_*i*_	Rank	*η*_*i*_	Rank	*η*_*i*_	Rank	*η*_*i*_	Rank
Beijing	0.484	2	0.562	2	0.598	5	0.508	1	0.636	1	0.837	2	0.394	5	0.873	1
Wuhan	0.468	3	0.213	10	0.257	13	0.247	10	0.475	2	0.306	8	0.251	12	0.15	10
Guangzhou	0.433	7	0.809	1	0.554	6	0.272	8	0.47	3	0.552	3	0.623	2	0.202	7
Shanghai	0.455	4	0.558	3	0.945	1	0.274	7	0.44	4	0.996	1	0.581	3	0.608	2
Nanjing	0.302	16	0.183	11	0.317	10	0.209	14	0.427	5	0.332	7	0.276	8	0.187	8
Zhengzhou	0.414	8	0.123	14	0.293	12	0.274	6	0.419	6	0.172	16	0.17	16	0.107	15
Shenzhen	0.829	1	0.423	5	0.631	2	0.368	5	0.416	7	0.387	6	0.751	1	0.389	3
Jinan	0.277	17	0.073	19	0.146	14	0.4	3	0.384	8	0.173	15	0.049	19	0.068	17
Lanzhou	0.373	9	0.026	21	0.01	21	0.423	2	0.383	9	0.003	21	0.031	20	0.001	21
Xi’an	0.364	10	0.219	8	0.097	18	0.191	16	0.38	10	0.174	14	0.159	17	0.182	9
Tianjin	0.274	18	0.219	9	0.617	4	0.242	12	0.37	11	0.46	4	0.358	6	0.358	5
Chengdu	0.438	6	0.309	7	0.397	9	0.135	20	0.332	12	0.3	10	0.265	10	0.216	6
Hangzhou	0.333	11	0.326	6	0.428	8	0.2	15	0.291	13	0.301	9	0.276	9	0.141	11
Chongqing	0.169	21	0.485	4	0.618	3	0.228	13	0.271	14	0.399	5	0.17	15	0.377	4
Xiamen	0.305	14	0.119	16	0.133	16	0.175	17	0.239	15	0.063	18	0.435	4	0.13	12
Urumqi	0.447	5	0.052	20	0.057	19	0.075	21	0.223	16	0.01	20	0.023	21	0.016	20
Qingdao	0.202	19	0.11	18	0.309	11	0.25	9	0.207	17	0.199	12	0.202	14	0.115	13
Dalian	0.314	13	0.146	13	0.128	17	0.171	18	0.202	18	0.149	17	0.26	11	0.057	19
Shenyang	0.304	15	0.123	15	0.145	15	0.243	11	0.2	19	0.195	13	0.293	7	0.061	18
Ningbo	0.321	12	0.159	12	0.435	7	0.398	4	0.158	20	0.204	11	0.24	13	0.114	14
Nanning	0.178	20	0.112	17	0.051	20	0.149	19	0.157	21	0.053	19	0.135	18	0.096	16
Mean	0.366		0.255		0.341		0.259		0.337		0.298		0.283		0.212	
Standard Deviation	0.141		0.204		0.249		0.107		0.125		0.252		0.190		0.210	
Range	0.660		0.783		0.935		0.433		0.479		0.993		0.728		0.872	
Coefficient of Variation	0.387		0.802		0.730		0.414		0.371		0.845		0.672		0.993	

*Data Source* “2022 China Urban Statistical Yearbook,” “2022 China Logistics Statistical Yearbook,” “2022 China Transportation Statistics Yearbook,” “2021 Civil Aviation Airport Production Statistical Bulletin,” calculated using the entropy-weighted TOPSIS analysis method.

**Table 3:** Competitiveness of aviation logistics industry clusters at the element layer for national logistics node cities.

City	Resource competitiveness		Facility competitiveness		Industry competitiveness		Innovation competitiveness		Market competitiveness		Government competitiveness	
	*η*_*i*_	Rank	*η*_*i*_	Rank	*η*_*i*_	Rank	*η*_*i*_	Rank	*η*_*i*_	Rank	*η*_*i*_	Rank
Beijing	0.899	1	0.653	1	0.737	1	1	1	0.656	3	1	1
Wuhan	0.823	2	0.452	6	0.672	2	0.676	4	0.844	1	0.839	2
Guangzhou	0.599	4	0.646	2	0.613	4	0.58	7	0.636	4	0.391	3
Shanghai	0.619	3	0.447	7	0.644	3	0.779	3	0.674	2	0.083	7
Nanjing	0.576	6	0.278	16	0.435	7	0.391	11	0.46	5	0.325	5
Zhengzhou	0.596	5	0.377	10	0.528	5	0.089	14	0.309	11	0.365	4
Shenzhen	0.421	9	0.357	11	0.304	13	0.797	2	0.309	10	0.041	10
Jinan	0.534	7	0.469	4	0.317	11	0.249	12	0.316	9	0.097	6
Lanzhou	0.42	10	0.516	3	0.213	17	0.433	10	0.179	17	0.064	9
Xi’an	0.431	8	0.253	20	0.282	15	0.624	5	0.34	7	0.069	8
Tianjin	0.384	13	0.307	15	0.312	12	0.594	6	0.187	16	0.019	15
Chengdu	0.419	11	0.31	14	0.375	8	0.132	13	0.324	8	0.036	11
Hangzhou	0.351	14	0.311	13	0.372	9	0.447	8	0.121	18	0.007	17
Chongqing	0.216	18	0.455	5	0.167	19	0.041	15	0.355	6	0.029	12
Xiamen	0.262	16	0.262	18	0.465	6	0.000	20	0.253	13	0.022	14
Urumqi	0.392	12	0.338	12	0.293	14	0.013	17	0.222	15	0.022	13
Qingdao	0.201	20	0.158	21	0.36	10	0.445	9	0.007	20	0.000	21
Dalian	0.228	17	0.278	17	0.24	16	0.01	19	0.29	12	0.005	18
Shenyang	0.279	15	0.383	9	0.119	20	0.000	21	0.112	19	0.015	16
Ningbo	0.161	21	0.384	8	0.035	21	0.025	16	0.005	21	0.000	20
Nanning	0.205	19	0.256	19	0.17	18	0.011	18	0.247	14	0.005	19
Mean	0.429		0.376		0.364		0.349		0.326		0.164	
Standard Deviation	0.203		0.126		0.190		0.320		0.220		0.280	
Range	0.738		0.495		0.702		1.000		0.839		1.000	
Coefficient of Variation	0.472		0.336		0.521		0.915		0.676		1.711	

*Data Source* “2022 China Urban Statistical Yearbook,” “2022 China Logistics Statistical Yearbook,” “2022 China Transportation Statistics Yearbook,” “2021 Civil Aviation Airport Production Statistical Bulletin,” calculated using the entropy-weighted TOPSIS analysis method.

**Table 4:** Competitiveness of subsystem layer and objective layer of aviation logistics industry clusters in national logistics hub cities.

City	Grounding competitiveness		Core competitiveness		Regulatory competitiveness		Competitiveness	
	*η*_*i*_	Rank	*η*_*i*_	Rank	*η*_*i*_	Rank	*η*_*i*_	Rank
Beijing	1.000	1	1.000	1	0.969	1	1.000	1
Wuhan	0.869	3	0.899	2	0.995	2	0.989	2
Guangzhou	0.889	2	0.472	3	0.931	4	0.865	3
Shanghai	0.696	4	0.408	4	0.959	3	0.780	4
Nanjing	0.532	7	0.103	12	0.557	6	0.332	5
Zhengzhou	0.330	8	0.238	8	0.610	5	0.314	6
Shenzhen	0.630	5	0.049	13	0.283	9	0.236	7
Jinan	0.269	9	0.398	5	0.261	11	0.172	8
Lanzhou	0.565	6	0.138	9	0.083	19	0.163	9
Xi’an	0.140	15	0.281	6	0.266	10	0.086	10
Tianjin	0.193	12	0.011	14	0.368	8	0.079	11
Chengdu	0.044	19	0.000	18	0.416	7	0.073	12
Hangzhou	0.157	14	0.238	7	0.171	16	0.053	13
Chongqing	0.264	10	0.001	15	0.173	15	0.043	14
Xiamen	0.137	17	0.134	10	0.197	12	0.034	15
Urumqi	0.209	11	0.000	16	0.175	14	0.031	16
Qingdao	0.002	21	0.131	11	0.143	17	0.015	17
Dalian	0.188	13	0.000	19	0.019	20	0.013	18
Shenyang	0.046	18	0.000	21	0.176	13	0.013	19
Ningbo	0.139	16	0.000	17	0.000	21	0.007	20
Nanning	0.027	20	0.000	20	0.094	18	0.003	21
Mean	0.349		0.214		0.374		0.252	
Standard Deviation	0.310		0.287		0.331		0.342	
Range	0.998		1.000		0.995		0.997	
Coefficient of Variation	0.890		1.340		0.886		1.356	

*Data source* “2022 China City Statistical Yearbook”, “2022 China Logistics Statistical Yearbook”, “2022 China Transport Statistical Yearbook”, “2021 Civil Aviation Airport Production Statistics Bulletin”, calculated using entropy-weighted TOPSIS method.

## Analysis of competitiveness at the variable and element layers

### Resource competitiveness

As can be seen from Tables 2 and 3, overall, the mean resource competitiveness of all cities is 0.429, with a range of 0.738, a standard deviation of 0.203, and a coefficient of variation of 0.472, indicating significant differences in resource competitiveness. Specifically, there are substantial differences in labor, capital, and land, while transportation location and economic foundation have smaller differences. Further analysis shows that Zhengzhou’s resource competitiveness (0.384) is 0.045 lower than the mean, ranking 13th, 0.515 lower than Beijing (0.899) in first place, and only 0.223 higher than Urumqi (0.161) in last place, indicating relatively low resource competitiveness. The reasons are that Zhengzhou’s labor (0.190), capital (0.129), and economic foundation (0.368) are all below the mean, indicating significant competitive disadvantages. However, land (0.329) and transportation location (0.690) are both above the mean, indicating a certain competitive advantage. Therefore, to improve its resource competitiveness, Zhengzhou must continually increase its investment in elements such as labor and capital, accelerate the transformation of its economic growth mode, and solidify its economic development foundation while fully utilizing its land and transportation location advantages.

### Facility competitiveness

As can be seen from Tables 2 and 3, overall, the mean facility competitiveness of all cities is 0.376, with a range of 0.495, a standard deviation of 0.126, and a coefficient of variation of 0.336, indicating relatively small differences in facility competitiveness. Specifically, there are significant differences in transportation and communication facilities, while ecological and living facilities have smaller differences. Further analysis shows that Zhengzhou’s facility competitiveness (0.307) is 0.069 lower than the mean, ranking 15th, 0.346 lower than Beijing (0.653) in first place, and 0.05 lower than Wuhan (0.357). This indicates relatively low facility competitiveness. The reasons are that aside from living facilities (0.414), which are above the mean, Zhengzhou’s communication (0.153), ecological (0.484), and transportation (0.296) facilities are all below the mean. Therefore, the key to enhancing Zhengzhou’s facility competitiveness lies in accelerating the construction of communication, ecological, and transportation facilities, providing a favorable environment for investment and business development for logistics enterprises, talent, and intermediary institutions.

### Industry competitiveness

As can be seen from Tables 2 and 3, overall, the mean Industry competitiveness of all cities is 0.364, with a range of 0.702, a standard deviation of 0.190, and a coefficient of variation of 0.521, indicating significant differences in Industry competitiveness. Specifically, there are substantial differences in logistics industry scale and auxiliary industries, while the differences in logistics industry advantages are relatively small. Further analysis reveals that the industry competitiveness of Zhengzhou (0.312) is 0.052 lower than the average, ranking 12th, 0.425 behind the first-ranked Beijing (0.737), and 0.277 higher than the last-ranked Urumqi (0.035). This suggests that the competitiveness of Zhengzhou’s logistics industry is relatively low. The cause of this lies in the fact that the scale of Zhengzhou’s logistics industry (0.123) and the support of auxiliary industries (0.293) are both below average, while the advantages of the logistics industry (0.274) are above average, ranking sixth. In-depth analysis shows that the advantages of the logistics industry (such as passenger throughput growth rate and cargo/mail throughput growth rate) represent future competitive potential, while the scale of the logistics industry (postal business revenue, express delivery volume, etc.) and the support of auxiliary industries (wholesale and retail industry employees, industrial output value of large-scale industries, etc.) represent current competitive advantages. This indicates that although Zhengzhou’s logistics industry currently lacks obvious competitive advantages, it possesses significant future competitive potential. Therefore, Zhengzhou must focus on expanding the scale of its logistics industry and improving the capacity of auxiliary industries to turn this potential into actual competitive strength.

### Innovation competitiveness

As shown in Tables 2 and 3, the average innovation competitiveness of all cities is 0.349, with a range of 1.000, a standard deviation of 0.320, and a coefficient of variation of 0.915, indicating significant differences in innovation competitiveness. Further analysis shows that Zhengzhou’s innovation competitiveness (0.594) is 0.245 higher than the average, ranking sixth, 0.406 behind first-ranked Beijing (1.000), and higher than surrounding cities such as Chengdu (0.249), Xi’an (0.433), Jinan (0.447), and Chongqing (0.089). This suggests that Zhengzhou has a relatively prominent competitive advantage in innovation competitiveness. The reason for this is that in recent years, a series of national strategies such as the Zhengzhou Airport Economy Comprehensive Experimental Zone, Zheng-Luo New Independent Innovation Demonstration Zone, Henan Free Trade Pilot Zone, and Henan Big Data Comprehensive Experimental Zone have been implemented in Zhengzhou, accelerating the pace of technological innovation, institutional innovation, and management innovation, and enhancing innovation competitiveness.

### Market competitiveness

As shown in Tables 2 and 3, the average market competitiveness of all cities is 0.326, with a range of 0.839, a standard deviation of 0.220, and a coefficient of variation of 0.676, indicating significant differences in market competitiveness. Specifically, there are substantial differences in local and external markets. Further analysis shows that Zhengzhou’s market competitiveness (0.187) is 0.138 lower than the average, ranking 16th, 0.657 lower than the first-ranked Shanghai (0.844), and only 0.182 higher than the last-ranked Urumqi (0.005). This suggests that Zhengzhou’s market competitiveness is relatively weak. The local market reflects the level of internal market exchange activity, which affects the scale and speed of the logistics industry cluster, while the external market reflects the frequency of external interactions, which impacts the prospects and demand of the logistics industry cluster. Both Zhengzhou’s local market (0.172) and external market (0.170) are below the average, indicating that the city’s market exchange activity and the frequency of external interactions are relatively low, which will limit the future scale and speed of its logistics industry cluster.

### Government competitiveness

As shown in Tables 2 and 3: overall, the mean government competitiveness of all logistics hub cities is 0.164, with a range of 1.000, a standard deviation of 0.280, and a coefficient of variation of 1.711, indicating significant differences in government competitiveness among the cities. Further analysis shows that Zhengzhou’s government competitiveness (0.019) is 0.145 lower than the mean, ranking 15th, with a difference of 0.981 compared to the top-ranked Beijing (1.000), and lower than neighboring Wuhan (0.041), Xi’an (0.064), and Chengdu (0.097). This suggests that Zhengzhou has a significant disadvantage in government competitiveness. Government support for the development of logistics industry clusters is not only reflected in planning and development, financial support, tax incentives, financial credit, and land supply, but also in clean and efficient administration, simplified procedures, and convenient services. To ensure the objectivity of the entropy weight assignment, only quantitative indicators were selected, while qualitative indicators were ignored, which may underestimate the government’s support for logistics industry clusters^29–31^.

### Proximity analysis of subsystem layer and objective layer

This research limited the data scope to 2021 and 2022 due to incompleteness and the impact of the COVID-19 pandemic on the data for 2019 and 2020. As shown in Table 4, the overall mean competitiveness of aviation logistics industry clusters for all cities is 0.252, with a range of 0.997, a standard deviation of 0.342, and a coefficient of variation of 1.356, indicating significant variation in competitiveness. Notably, the differences in infrastructural and regulatory competitiveness are relatively small, while the disparities in core competitiveness are substantial. This finding suggests that the competitiveness of aviation logistics industry clusters in all cities hinges primarily on their core competencies. Further analysis revealed that Zhengzhou’s aviation logistics industry cluster (0.053) falls 0.199 below the mean competitiveness, ranking 13th out of 21 cities. This indicates that Zhengzhou holds a relatively modest competitive advantage in the aviation logistics industry. The primary factor contributing to this is the sub-standard performance of Zhengzhou in subsystems such as infrastructure (0.157) and regulation (0.171), compared to the mean. On the other hand, Zhengzhou’s core competitiveness (0.238) surpasses the mean, with innovation competitiveness outperforming the mean and industry competitiveness falling short. This suggests that Zhengzhou’s aviation logistics industry cluster’s competitiveness hinges primarily on the performance of its core competencies, while the lower performance of subsystems like infrastructure and regulation significantly hinders overall competitiveness. To enhance Zhengzhou’s competitive standing, it is crucial to bolster the competitiveness of elements such as resources, facilities, markets, government, and industry, with a focus on elevating industry competitiveness as the central element.

## Results and discussion

By constructing an aviation logistics industry cluster competitiveness GKA evaluation model and taking 21 national logistics node cities as evaluation objects, the entropy-weighted TOPSIS method was applied for evaluation research. The competitiveness of Zhengzhou aviation logistics industry cluster is below average, mainly due to the low competitiveness of subsystems such as infrastructure and regulation. The deeper reasons are the relatively low competitiveness of factors such as resources, facilities, markets, government, and industry. Therefore, the key to improving the competitiveness of the Zhengzhou aviation logistics industry cluster is to enhance the competitiveness of factors such as resources, facilities, markets, and government, with the core being the promotion of industry competitiveness. Accordingly, the following recommendations are proposed:

### Strengthen the input of production factors and consolidate the economic foundation of the aviation port area

First, strengthen the introduction and training of aviation logistics talent. Encourage international logistics exchanges and cooperation, incentivize logistics enterprises to build platforms for attracting outstanding aviation logistics talent; promote cooperation between port area logistics enterprises and universities for joint cultivation of fourth-party logistics operation talent; and improve the internationally-aligned logistics talent certification mechanism to enhance the professional qualification certification of logistics enterprise employees. Second, increase credit support for logistics enterprises. Attract national and foreign banks to settle in the port area and cooperate with local banks in equity and business. Allow legal persons or individuals to invest in local financial institutions; introduce funds from outside the province through syndicated loans and joint loans to increase credit support for logistics enterprises, and use fiscal subsidies to guide credit, private and foreign capital investment in port area logistics facilities, thereby achieving diversified investment in the logistics industry. Develop internet finance vigorously to improve the efficiency of logistics enterprises’ fund settlement and accelerate the updating of logistics equipment.

### Strengthen infrastructure construction and build a public logistics information platform

First, strengthen infrastructure construction. Enhance the construction of port area stations, warehousing facilities, international and national cargo airline networks, ground transportation networks, communication facilities, and platforms; improve public facilities such as living, medical, ecological, and leisure to create a comfortable environment for high-end business professionals. Second, build a public logistics information platform. Drawing on the experience of Ningbo’s “Fourth-Party Logistics Platform”, the Zhengzhou municipal government should lead, with internet companies initiating and logistics enterprises participating, to jointly establish a public logistics information platform. Enterprises should establish logistics information management systems and achieve data sharing with public logistics information platforms and e-government systems. Accelerate the development of internationally-aligned generic logistics information standards.

### Expand the scale of the logistics industry and enhance the supporting capacity of auxiliary industries

Firstly, expanding the scale of the logistics industry: To enhance the ability of logistics enterprises to withstand risks, mergers, acquisitions, and reorganizations of logistics enterprises can be promoted. The government can attract the headquarters or operation centers of well-known national and foreign logistics companies to settle in the port area, and encourage local enterprises to form alliances with them through joint ventures, cooperation, and shareholding. The government can also accelerate the innovation of logistics service models, facilitate information sharing among small and medium-sized enterprises, and meet the increasingly diverse logistics demands. The construction of aviation logistics parks can also be expedited, and logistics enterprises and high-tech enterprises can be attracted to settle in by offering financial support and policy incentives, forming a complete aviation logistics industry chain. Secondly, enhancing the supporting capacity of auxiliary industries: To accelerate the development of logistics industry clusters, follow-up services for enterprises settled in the port area can be strengthened, and more related enterprises can be attracted to settle in. The government can expedite the development of high-end exhibitions, aviation insurance, aviation finance, cross-border e-commerce, and health and leisure modern service industries. A global commodity procurement and trading platform integrating modern logistics, e-commerce, international trade, and financial services can be established to create a fair and honest market competition environment. By expanding the scale of the logistics industry and enhancing the supporting capacity of auxiliary industries, the competitiveness of the aviation logistics industry cluster in Zhengzhou can be enhanced. These measures can help to attract more investment, improve logistics infrastructure, and promote the development of the aviation logistics industry. By doing so, Zhengzhou’s aviation logistics industry cluster can better compete with other clusters within China and globally.

### Establish an innovative development platform and continuously promote innovation-driven development

From a macro perspective, guided by government investment and with enterprises as the main body, establish innovative platforms such as key laboratories, engineering technology research centers, and public aviation-oriented research institutions. Guide key universities and research institutes to set up branches or R&D centers in the port area, vigorously support key technology intermediary organizations and technology-based incubators, and enhance independent research and development capabilities. From a micro perspective, encourage technology innovation of leading enterprises in the port area and implement investment strategies such as “innovation chain,” “industry chain,” “financial chain”, and “policy chain” to attract innovative high-tech small and medium-sized enterprises to settle in, forming healthy competition and cooperative relationships.

### Expand logistics market demand and promote the development of logistics industry clusters

First, improve the local market consumption level. The expansion of the logistics market stems from the increase in residents’ consumption levels. Therefore, accelerate the development of the service industry, increase employment opportunities for residents, raise residents’ income, encourage credit consumption, and improve local market consumption levels. Second, strengthen external communication and cooperation in key areas such as finance, exhibitions, tourism, culture, talent, technology, planning, education, and health to increase demand in external markets. Finally, focus on cultivating five airport-related industry clusters: aviation logistics, aviation industry, high-tech product manufacturing, international business exhibitions, and health and leisure industries.

### Construct four major service systems and comprehensively improve the level of government services

Firstly, building four major service systems: The first service system should focus on promoting investment liberalization, trade facilitation, and regulatory rule of law. It should establish an efficient, business-friendly, one-stop government service system. The second service system should focus on building a multi-modal transport, global logistics, and unified customs inspection regulatory service system. The third service system should focus on building a diversified financing, efficient service, and integrated financial service system. The fourth and final service system should focus on building a sound, mediation-integrated, and equal legal service system. By building these service systems, the government can create a more supportive environment for the aviation logistics industry, which can lead to enhanced competitiveness.

Secondly, comprehensively improving the level of government services: To promote technology intermediation and consulting services, intermediary organizations such as banks, insurance, accounting, legal, evaluation, and advertising agencies can be introduced. Financing channels can be broadened by leveraging the preferential policies of the Zhengzhou Airport Zone’s pilot program, implementing financial subsidies, technological reforms, tax reductions and rate adjustments, and promoting policies suitable for the development of the airport industry. The marketization of logistics can be promoted by relaxing business licensing and registration requirements, providing policy preferences in land, tax, and finance, and improving logistics market access, supervision, and exit mechanisms. These measures can help to improve the level of government services and create a more favorable business environment for the aviation logistics industry. By building four major service systems and comprehensively improving the level of government services, the competitiveness of the aviation logistics industry cluster in Zhengzhou can be enhanced. These measures can help to attract more investment, improve logistics infrastructure, and promote the development of the aviation logistics industry. By doing so, Zhengzhou’s aviation logistics industry cluster can better compete with other clusters within China and globally.

## Conclusion

In conclusion, this study has evaluated the competitiveness of the aviation logistics industry cluster in Zhengzhou, China, using the GKA evaluation model and the entropy-weighted TOPSIS method. The results show that Zhengzhou’s aviation logistics industry cluster is moderately low in competitiveness due to the weak competitiveness of its foundational and regulatory subsystems. Based on the findings, the study recommends that efforts be made to improve the competitiveness of key elements such as resources, facilities, markets, and government, with a particular focus on elevating industry competitiveness, followed by the development of appropriate regulatory strategies. By doing so, Zhengzhou’s aviation logistics industry cluster can enhance its competitiveness and better compete with other clusters within China and globally. The study also found significant differences in the competitiveness of aviation logistics industry clusters in all cities, mainly depending on their core competitiveness. Further analysis showed that Zhengzhou’s competitiveness was relatively low compared to other cities, mainly due to the lower competitiveness of elements such as resources, facilities, markets, government, and industry. Therefore, the key to improving the competitiveness of Zhengzhou’s aviation logistics industry cluster is to enhance the competitiveness of these elements, with the core being the improvement of industry competitiveness. Overall, the findings of this study provide valuable insights for policymakers and industry stakeholders seeking to enhance the competitiveness of aviation logistics industry clusters in China.

It should be noted that the current research did not use a limiting indicator that would doubt the generality of its application. For this reason, it seems that it can be used in other geographical areas. Nevertheless, to ensure the applicability of the proposed indicator model in evaluating the aviation logistics industry competitiveness in broader scenarios, this issue can be studied in future works.

## Data Availability

All data generated or analyzed during this study are included in this published article.
